# Optical computing metasurfaces: applications and advances

**DOI:** 10.1515/nanoph-2023-0871

**Published:** 2024-02-13

**Authors:** Hongqiang Zhou, Chongli Zhao, Cong He, Lingling Huang, Tianlong Man, Yuhong Wan

**Affiliations:** School of Physics and Optoelectronic Engineering, Beijing University of Technology, Beijing 100124, China; Beijing Engineering Research Center of Mixed Reality and Advanced Display, School of Optics and Photonics, Beijing Institute of Technology, Beijing 100081, China

**Keywords:** metasurface, optical computing, functional device

## Abstract

Integrated photonic devices and artificial intelligence have presented a significant opportunity for the advancement of optical computing in practical applications. Optical computing technology is a unique computing system based on optical devices and computing functions, which significantly differs from the traditional electronic computing technology. On the other hand, optical computing technology offers the advantages such as fast speed, low energy consumption, and high parallelism. Yet there are still challenges such as device integration and portability. In the burgeoning development of micro–nano optics technology, especially the deeply ingrained concept of metasurface technique, it provides an advanced platform for optical computing applications, including edge detection, image or motion recognition, logic computation, and on-chip optical computing. With the aim of providing a comprehensive introduction and perspective for optical computing metasurface applications, we review the recent research advances of optical computing, from nanostructure and computing methods to practical applications. In this work, we review the challenges and analysis of optical computing metasurfaces in engineering field and look forward to the future development trends of optical computing.

## 1 Introduction

Due to the rapid growing of information, various challenges are raised, involving massive data storage and real-time processing. Specifically, the demand for real-time processing of diverse data types has evolved into a standard configuration in devices across various industries, such as image edge detection [1], [2], [3], [4], [5], [6], image recognition [7], [8], [9], [10], [11], [12], [13], logic operation [14], [15], [16], [17], [18], [19], [20], and space-time coding communication [21], [22], [23], [24]. And artificial intelligence, with deep learning [25] at its core, is propelling human society into the era of intelligence. Computing power plays a crucial role in driving the development of artificial intelligence. However, the disparity between the computing power supplied by traditional electronic computing and the exponentially increasing computing demands generated by artificial intelligence is widening. For example, the matrix–matrix multiplication and the addition operations commonly used in deep learning will generate huge data access requirements when running in traditional computing systems. This underscores the pressing need to identify novel avenues for computing power growth to address the escalating demands of the intelligent age. The current data processing is mostly carried out by integrated electronic circuits and program algorithms. Although electronic computing has been quite mature, it still has many unavoidable defects, including high ohmic loss and power consumption, lower coding response speed, huge computing system size, and low integration. Furthermore, semiconductor- and metal-based electronic components face limitations in speed improvement, as the size parameters of fabricated units have reached the quantum limit. Hence, the fabrication of electronic chips characterized by enhanced performance and increased transistor density is poised to confront an inevitable bottleneck that demands careful consideration.

**Figure 1:**
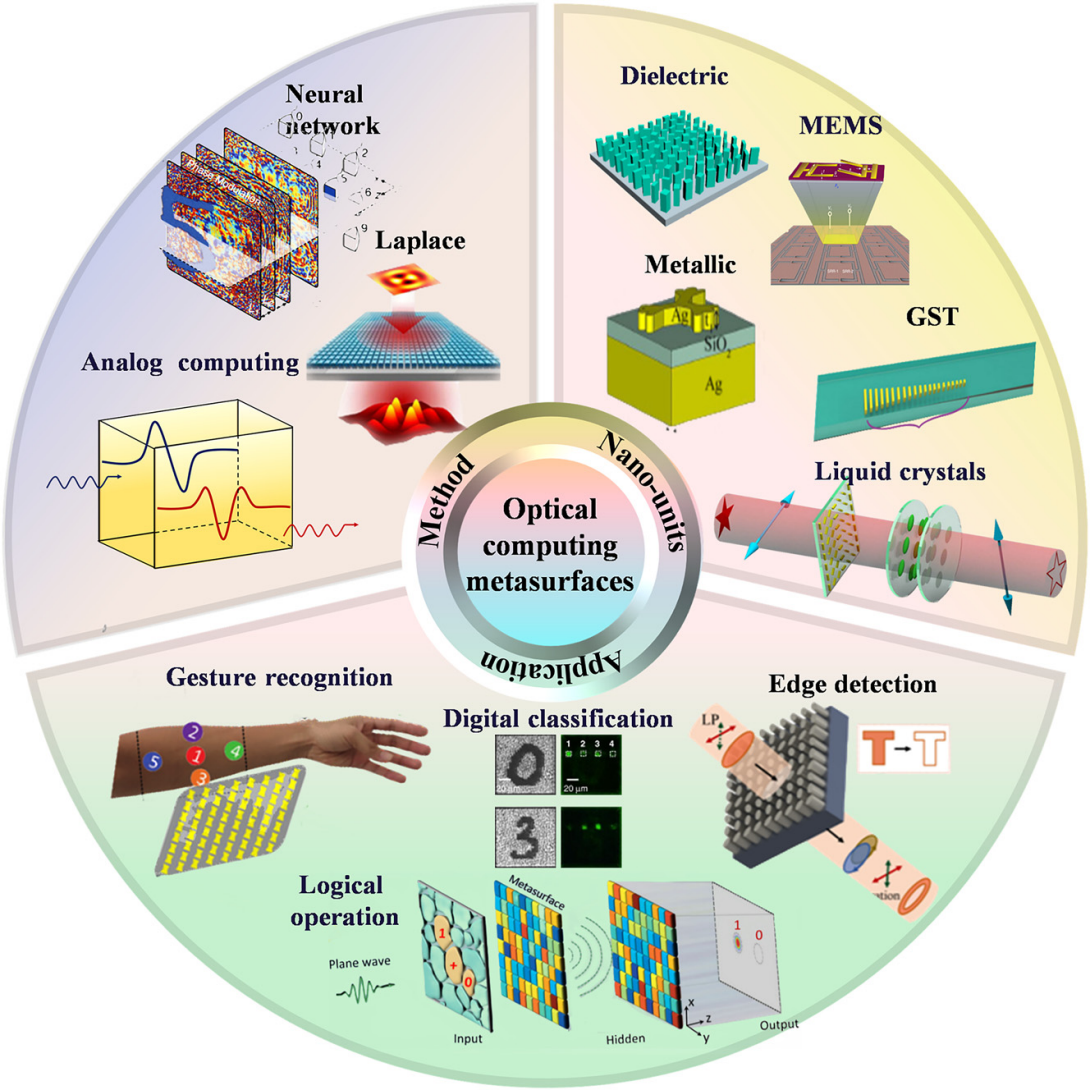
Optical computing metasurfaces: from principle to applications and advances. Reproduced with permission [63], [64], [65], [66], [67], [68], [69], [70], [71], [72].

Consequently, the research of alternative computing elements and modes has become a breakthrough to solve these problems. This underscores the pressing need to identify novel avenues for computing power growth to address the escalating computation demands. In addressing the challenge of insufficient computing power, optical computing has attracted wide attention due to its unique advantages of high parallelism, low power consumption, and high response speed [26], [27], [28], [29], [30]. Optical computing is emerging as a promising alternative, as it not only offers faster computational speeds but also extremely lower energy consumption and massive parallelism. Drawing on the interaction between light and matter, optical computing can be effectively realized through a carefully crafted design [31], [32]. The implementation of optical artificial neural networks provides new solutions for various applications of optical computing. It is worth mentioning that the optical computing technology based on optical neural networks exhibits a high delay bandwidth product in optical information transmission, where different wavelengths of light can cross each other without interfering. With the rapid advancement of micro–nano optics devices, particularly metasurfaces, new avenues are opening up for modulating optical fields, processing optical information, and optical computing [33], [34]. It is expected to solve the inherent problems of the traditional optical systems so that the optical computing can be lighted, integrated, and widely promoted.

To facilitate understanding, we firstly introduce the basic modulation principles of metasurface. Then, we will discuss the optical computing research advances based on metasurfaces in recent years. Furthermore, from the perspective of metasurface modulation principle and applications, different metasurfaces consist of nanostructure components (fundamental materials) and the worldwide applications in optical computation are introduced in image edge detection, digital or image classification, logic computation, and on-chip optical computing. Additionally, this paper reviews the advancements and challenges of various optical computing metasurfaces. And it further looks forward to potential developments and engineering research directions by applying optical computing metasurfaces (Figure 1).

## 2 The basic modulation principle of metasurfaces

Metasurface is the two-dimensional equivalent of metamaterial, which is discrete subwavelength structures composed of artificial meta-atoms [35], [36], [37], [38], [39], [40], [41], [42], [43]. By designing the geometry, material(composition), rotation angle, and arrangement of meta-atoms, it can be enhanced to realize the control of the basic properties of light, including amplitude [44], [45], [46], [47], phase [48], [49], [50], polarization [51], [52], [53], [54], [55], frequency [56], [57], orbital angular momentum (OAM) [58], [59], [60], [61], [62], etc.

### 2.1 Phase control

The phase modulation of the metasurface is based on the discontinuous phase modulations caused by the mechanism of light interacts with matter. Abrupt phase change can be tailored by changing geometrical parameters such as shape, size, and rotation. The key to phase modulation is to achieve a range of phase regulation from 0 to 2π to reconstruct wavefronts. According to its physical mechanism, main phase control can be divided into the resonance phase, propagation phase, and geometry phase. The principle of resonance phase is based on the internal resonance phenomena nanostructure to modulate electromagnetic waves. When the electromagnetic wave illuminates the nanostructure, the resonance phenomena of the electromagnetic waves change its phase and amplitude [73]. By adjusting the microstructure parameters or materials, the reflection, transmission, and absorption can be controlled.

When light propagates in the nanostructure of the metasurface, the accumulated phase is also called the propagation phase [74]. The effective refractive index *n*_eff_ can be modulated by adjusting the transverse dimensions of the structures in the metasurfaces. The cumulative phase shift Δ*φ* of a beam passing through a nanostructure with a height of *h* is:

(1) $$\Delta \varphi =\frac{2\pi }{\lambda }n_{\text{eff}}h,$$

where *λ* is the wavelength in the free-space. Therefore, the propagation phase can be achieved by modulating the unit cell period and nano-antenna physical parameters [75].

As circular polarization light propagates through a dipole, its cross-polarization will carry additional phases, also called Pancharatnam–Berry (PB) phase [76]. The process of light through anisotropic nanostructures that have different responses to the two orthogonal electric field components *E*_*x*_ and *E*_*y*_ of incident circularly polarized light. The evolution path of incident light polarization can be controlled by changing the rotation angle of the anisotropic nanostructures to obtain additional phases. Therefore, the PB phase is only related to the rotation angle of the nanostructure. When a left-circularly or right-circularly polarized beam normally impinges onto the metasurface, the cross-polarized output case can be written as

(2) $$E_{\text{out}}=J\left(\theta \right)\cdot E_{L}=E_{R}\exp\left(-i2\theta \right)$$

where $E_{L}=\left(e_{x}+ie_{y}\right)/\sqrt{2}$ and $E_{R}=\left(e_{x}-ie_{y}\right)/\sqrt{2}$ . $J\left(\theta \right)$ is rotation Jones matrix. This process introduces an additional spin-independent phase *Φ*_*G*_ = −2*σθ*. Therefore, by controlling the local direction of the optical axis, any desired phase can be obtained, and the PB phase is only related to the geometric path of the system evolution, independent of the wavelength. The additional phase delay is equal to twice the rotation angle (*θ*) [77].

### 2.2 Amplitude control

The amplitude of the metasurface is mainly controlled by the transmissivity and reflectivity of nano-antenna modulation. The diffraction of light through apertures is produced through the superposition of an infinite number of secondary point sources, in according to the Huygens–Fresnel principle:

(3) $$U\left(P\right)=C\underset{\sum }{\iint U\left(P_{0}\right)K\left(\theta \right)}\frac{e^{\mathrm{jkr}}}{r}ds$$

where ∑ is a wavefront and *U*(*P*_0_) is the complex amplitude of any point *P*_0_ on the wavefront. And *r* is the distance from *P* to *P*_0_, and *θ* is the angle between $\bar{P_{0}P}$ and the normal of the elementary wave plane passing through *P*_0_. Gray modulation can also be achieved by changing the energy transit ratio. By optimizing the distribution of point sources, the required map can be designed at the location of the image [78]. Amplitude-type metasurfaces are widely used in areas such as holography and focusing because of their long working wavelength, flexible design, and simple processing [79], [80].

### 2.3 Polarization control

The key to polarization control is the different polarization (linear or circular) response of anisotropic nanostructures. By controlling the corresponding eigenmodes of orthogonal polarization separately, different polarized light can obtain different transmission or reflection efficiency at a broadband frequency, while carrying different phase delay of Δ*φ*. Furthermore, metasurface can realize the polarization control of light and can be applied to polarimetry [81], [82], polarization conversion [83], [84], and polarization imaging [85].

### 2.4 Frequency control

The structure size in the metasurface is subwavelength. When light passes through nanostructures, local energy resonance is significantly enhanced due to strong localization. The resonance response of nanostructures often varies with different light frequencies. The optimized structure array can be used for multifrequency, such as hyperspectral imaging. Otherwise, such strong interaction can also improve the efficiency of nonlinear optical effects, such as second harmonic generation (SHG) and third harmonic generation (THG), so metasurfaces can achieve nonlinear optical responses with different characteristics at specific wavelengths.

## 3 Advances of optical computing metasurfaces

Amidst the explosive growth of visual, auditory, tactile, and other types of information in the world, the demand for high-speed transmission and high-performance processing of large data capacity information is becoming increasingly urgent. Electronic circuits that traditionally incorporate a significant number of active devices are often used to process these data and perform mathematical calculations [86], but the inherent semiconductor properties of electronic devices limit the speed of calculation. Besides, the circuits themselves are also confront the defect of power consumption. In order to solve the bottleneck problem of electrical computing, optical computing is a very competitive candidate [87], [88], [89], [90], [91]. Compared with electrical devices, optical devices have a high degree of parallel characteristics, which can expand the processing data capacity by means of wavelength, polarization, mode, phase, etc. Meanwhile, due to the development of micro–nano fabrication technology, the size of optical devices can be reduced to the nanometer level, which provides a new scheme for the development of optical computing. With the continuous research in large-area processing techniques [92], [93], [94], such as nanoimprint lithography [95], holographic lithography [96], and self-assembly [97], the structural processing units of micro–nano devices are becoming smaller and more numerous. Hence, the large-area processing technology endows metasurfaces with more powerful potential applications in optical computing, optical information processing, and other related fields. As one of the most promising candidates for artificial structures, metasurfaces have attracted much attention because of their ability to modulate multiple freedom of incident electromagnetic waves, such as the amplitude, phase, polarization, frequency, and so on [98]. The metasurface can exhibit precise control over the phase and amplitude of light waves through the meticulous design of its microstructures. This property provides metasurfaces with a distinctive advantage in modulating and manipulating optical signals, aligning with the requirements of optical computing. The design flexibility of metasurfaces is remarkably high, enabling them to meet the varied requirements of optical computing tasks. Due to its composition of microstructures, a metasurface typically exhibits a lighter weight compared to conventional optical components. Metasurfaces can also be effectively integrated with other optical and electronic components to from compact systems. This approach is crucial for the integration and miniaturization of optical computing systems. Building upon the distinctive advantages of metasurfaces and their strong compatibility with optical computing, metasurfaces possess the attributes to serve as an excellent platform for optical computing. As with electronic computing, metasurfaces optical computing can be divided into two categories: digital computing and analog computing [98], [99], [100]. In optical digital calculation, a single basic logic operation includes AND, OR, NOT, XNOR, XOR, and NAND [63], [101], [102], as well as complex combined logic functions, such as total addition and total subtraction [103] and encoder [104]. In optical simulation calculations, once the input analog signal is converted into an optical signal, the high-dimensional optical control capabilities of micro–nanostructures can be harnessed to directly modulate the parameters of the input optical field.

### 3.1 The nano-units of optical computing metasurfaces

#### 3.1.1 Dielectric nano-antenna

Dielectric metasurfaces are typically constructed using materials with lower electrical conductivity and reduced losses, resulting in reduced energy losses in the optical and microwave frequency ranges. This renders dielectric metasurfaces particularly advantageous in optical computing applications demanding high efficiency and transmission. The dielectric metasurface is widely used in optical computing because of its high refractive index, low internal loss, and high transmission efficiency.

**Figure 2:**
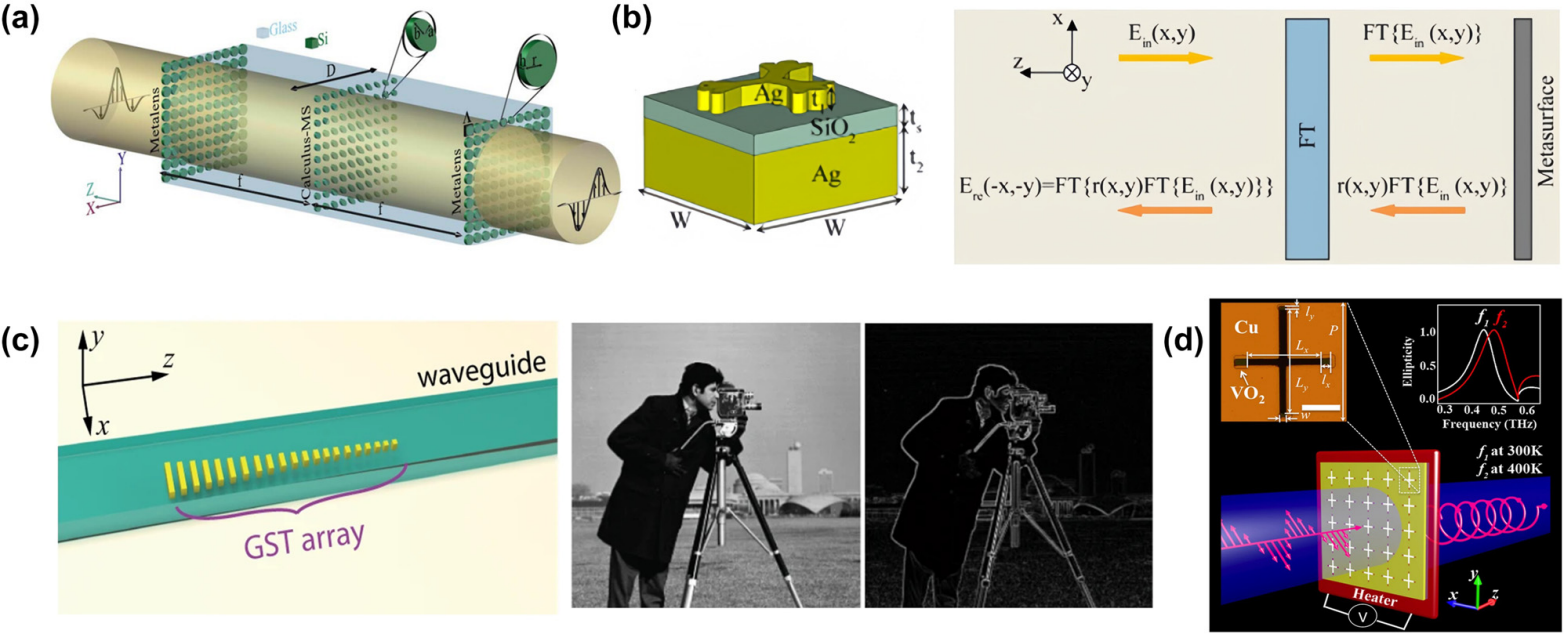
Basic modulation structure units. (a) Schematic diagram of the all-dielectric metasurface equation solver. (b) Schematic of performing a mathematical operation for a reflective metasurface. (c) Operation procedure of using the OCNN to recognize handwriting numbers from the MNIST database. The OCNN consists of a convolution layer with two kernels, a pooling and a fully connected layer. The output gives the answer whether the input image is “1” or “2.” (d) Schematic view of switchable THz quarter-wave plate design. The top left inset is a zoom image of the unit cell in the fabricated sample (*P* = 150, *L*_*x*_ = 90, *L*_*y*_ = 124, *l*_*x*_ = 9, *l*_*y*_ = 5, and *w* = 9 μm, and the scale bar is 50 μm). The top right inset is the simulated ellipticities of the output THz waves. (a) Reproduced with permission [106]. Copyright 2017, Optica Publishing Group. (b) Reproduced with permission [64]. Copyright 2017, Optica Publishing Group. (c) Reproduced with permission [65]. Copyright 2021, Nature Publishing Group. (d) Reproduced with permission [50]. Copyright 2015, Nature Publishing Group.

A. Chizari et al. propose a dielectric reflection meta-reflect-array (MRA) that overcomes the large volume problems associated with optical metamaterials and also demonstrates several structural advantages [105]. The meta-array supports Mie co-oscillation of the electric dipole and Mie resonance of the magnetic dipole, as well as multiple reflection patterns within the spacer layers, and thus enables full spatial control of the amplitude and phase distribution of the reflected cross-polarized light by changing the size of the resonator. By controlling the amplitude and phase distribution of the reflected cross-polarized light and simulating the reflection calculation system, the optical simulation computation operation is realized. Abdollahramezani et al. proposed an equation solver based on cascaded all-dielectric optical computing metasurface [106]. The structure diagram is shown in Figure 2(a). The phase and amplitude of the incident light wave can be controlled independently by changing the long and short axis of the nano-silicon ellipse structure. On this basis, the near-infrared (NIR) ordinary differential equation and the integral-differential equation solver are designed.

#### 3.1.2 Plasmon or metallic nano-antenna

There are a large number of freely moving electrons inside the metal, and these electrons dominate the electromagnetic properties of the metal under the action of the external electric field. Free electrons in metals can be coupled to incident light at the interface between metal and medium and produce surface plasmons [107]. In 2017, Zhu et al. realized spatial optical simulation differential operation in a small angle range [108]. A double-layer metal grating based on surface plasmon is proposed to achieve simultaneous differentiation in both temporal and spatial domains of optical signals. By using the unidirectional excitation of the surface plasmon polaritons (SPP) of the asymmetric metal grating, the vertical incident spatial and temporal differentiation are realized [109]. In addition, using the free control of the amplitude and phase of the light wave by metal–insulator–metal (MIM) metasurface, spatial simulation calculators are proposed and their operation capability is tested experimentally [99]. The MIM metasurface utilizes gap-surface plasmon mode to achieve independent amplitude and binary phase control of the reflection coefficient of 800 nm NIR light. The spatial simulation optical differentiator and integrator are designed and their computational functions are verified. The fused quartz spacer on top of the silver dendritic structure and the metasurface of the quasiperiodic silver dendritic substrate are proposed [64], the structure of which is shown in Figure 2(b). The phase of the common polarized light reflected by changing the shape of the silver dendritic structure is distributed spatially. The system consists of a Fourier transforming (FT) block and a reflective metasurface with a position-dependent reflection coefficient *r*(*x*, *y*). The metasurface green and red bands are differentially operated and the metasurface-based differential processor can be used for real-time edge detection and image contrast enhancement.

#### 3.1.3 Reconfigurable nano-antenna

Phase change material (PCM) [110], [111] refers to a class of functional optical materials that undergo structural phase change under the action of external fields (such as force, heat, light, electricity, et al.). This unconventional physical property is caused by the bonding mechanism changes [112]. PCM is accompanied by significant optical constant changes (Δ*n* > 1, Δ*k* produces orders of magnitude changes). Phase change materials commonly used in optical frequency bands include VO_2_, Ge_2_Sb_2_Te_5_ (GST) [113], [114], and polyvinyl alcohol (PVA) [93], [115].

GST is composed of germanium (Ge), antimony (Sb), and tellurium (Te) alloys. The phase change materials can transition from a disordered amorphous state to an ordered crystalline state through a heat-quench cycle [116]. In the infrared region, GST has a high refractive index in both crystalline and amorphous phases, indicating that the GST nanostructure should be able to support strong resonances closely related to its material phase [117], [118], [119]. Chalcogenide phase change materials have also become an example of achieving a nonvolatile, all-optical dielectric metasurface, which exhibits a low-loss Mie resonance in contrast to precious metals [120], [121].

Wu et al. proposed a multimode photon computing core based on an array of programmable mode converters [65]. The structure of the multimode photon computing core, as shown in Figure 2(c), is constructed with waveguides on a metasurface of phase-change materials. The programmable converter uses the refractive index change of the phase change material GST during the phase change to control the waveguide spatial mode with very high accuracy in mode contrast up to 64 levels. A metasurface optical computing system is constructed to perform image processing and recognition tasks with high precision, as shown on the right side of Figure 2(c). The system has a wide operating bandwidth and a small volume and is expected to be applied to photonic neural networks with ultra-high computational throughput. Based on the ability of the GST metasurface to regulate the light field, this structure will also become one of the important solutions for future optical computing.

Vanadium dioxide (VO_2_), as a reliable reconfigurable metasurface material, has also received extensive attention. VO_2_ exhibits an insulator–metal transition (IMT) near room temperature (*T*_IMT_ ≈ 67 °C in bulk crystals) [122], [123], [124]. It exhibits refractive index changes over a wide spectral range. The variation in VO_2_ properties stems from the change in its crystal structure [125], [126]. VO_2_ has a monoclinic lattice with the electrons localized in the atomic bonds at low temperatures. Compared with chalcogenide materials, their phase transition temperature is lower and they are easily stimulated by heat, electricity, and light.

By loading VO_2_ components on metasurface structures, an ultrathin switchable THz quarter wave plate (QWP) with a switching range of 34 GHz was experimentally demonstrated in Figure 2(d) [50]. It shows a schematic of the switchable QWP, which is composed of ultrathin asymmetric cross-shaped resonator arrays. VO_2_ pads are inserted at the end of the cross-shaped resonators. A linear normal incident THz wave polarized at *θ* = 45° to the two slots is converted into a circularly polarized light. The inserted VO_2_ is able to change the effective length of the metal resonators through the phase transition controlled.

#### 3.1.4 Programmable electric-control nano-antenna (FPGA/MEMS)

**Figure 3:**
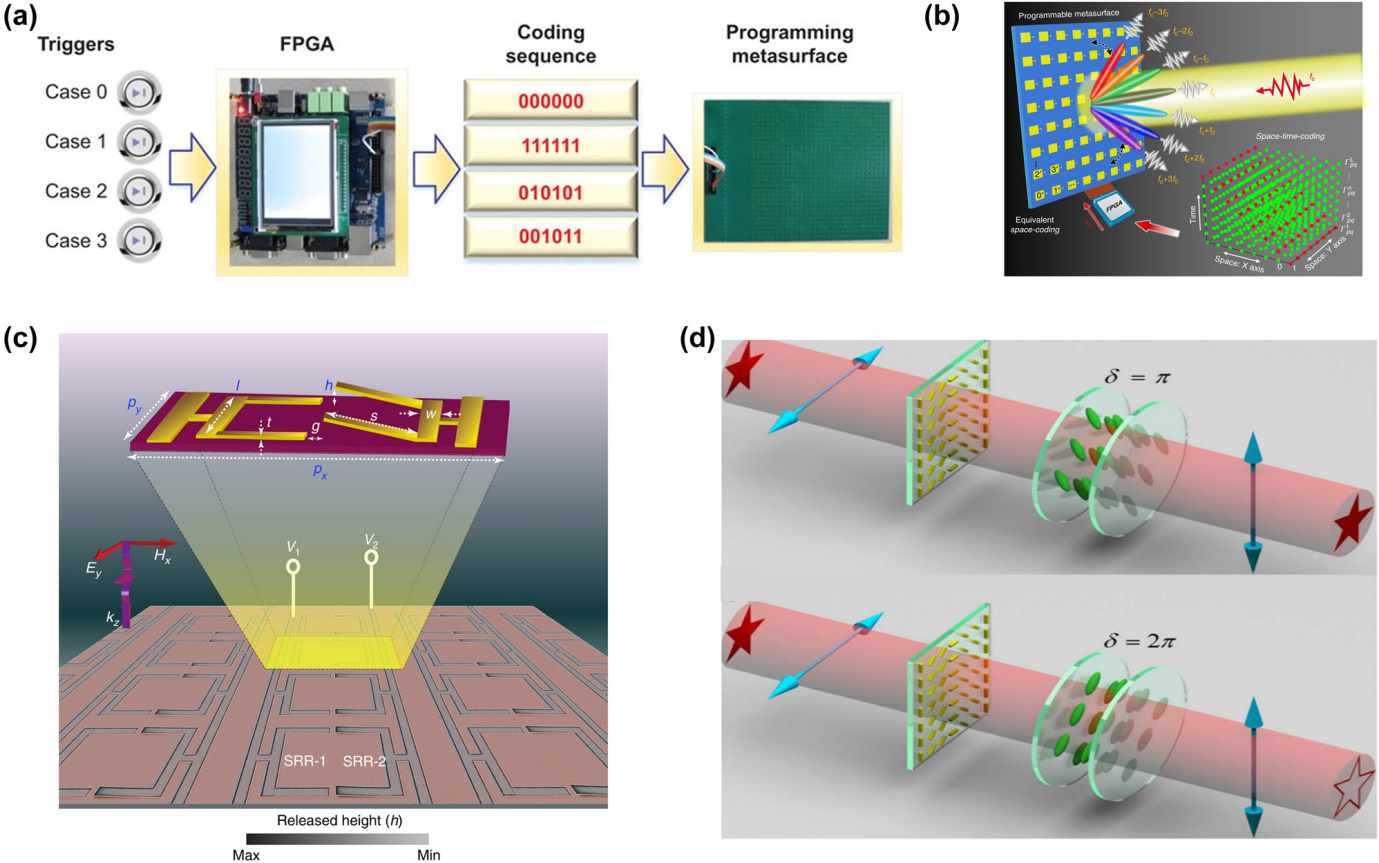
Electronic control structure units. (a) A flow diagram for realizing a programmable metasurface controlled by the FPGA. (b) Conceptual illustration of a space-time-coding digital metasurface. The increasing need for dynamic manipulation of light in modern optical systems is driving the development of electrically and optically tuned reconfigurable metasurfaces. (c) Colored scanning electron microscope (SEM) image of the MEMS Fano metasurface. (d) Realization of switch between bright-field image and edge image by tunable optical differentiator composed of metasurface and liquid-crystal phase plate. (a) Reproduced with permission [127]. Copyright 2014, Nature Publishing Group. (b) Reproduced with permission [128]. Copyright 2018, Nature Publishing Group. (c) Reproduced with permission [63]. Copyright 2018, Nature Publishing Group. (d) Reproduced with permission [72]. Copyright 2022, Optica Publishing Group.

In 2014, Cui’s team proposed a programmable metasurface, the unit is composed of a passive metasurface structure and a switching diode [127]. The switching diode has “on” and “off” states, and the reflected phase of the unit in the two states has a phase difference of 180°, thus forming a 1 bit phase code. Moreover, a Field Programmable Gate Array (FPGA) is used to provide the control voltage, such as high and low levels corresponding to the code “1” and “0,” respectively. As shown in Figure 3(a), the preset coding sequence is then input to different units on the metasurface via different PINs by the FPGA, thereby dynamically changing the digital coding pattern and realizing programmable control of different electromagnetic functions.

Additionally, the concept of spatiotemporal coded digital metasurface was proposed based on digital coding and programmable metasurface [128]. As shown in Figure 3(b), a two-dimensional array composed of *M* × *N* programmable basic units. Each unit is integrated with adjustable devices whose amplitude, phase, and polarization can be controlled in real time. The external control FPGA based on programmable devices can simultaneously output *M* × *N* control signals to control each unit of the metasurface, where each signal is a specific coding sequence that varies periodically in real time. As shown to the right of Figure 3(b), the green and red dots represent the coding states “0” and “1,” respectively.

MEMS can accurately generate a certain external force (Coulomb force, amperage force, etc.) on the target structure through an external electric field, magnetic field, thermal stimulation, and other ways on the micro/nano-scale and quantitatively deform its geometry by breaking the original mechanical balance of the metasurface, so as to accurately and dynamically regulate its optical response. The excitation and active tuning of sharp Fano resonances are proposed on MEMS reconfigurable metasurface that exhibits multiple-input–output (MIO) properties in both near and far-field optical properties [63]. The reconfigurable geometry of the MEMS Fano-metasurface provides various structural meta-stable states by using two independently controllable electrical inputs and an optical/near-field readout that enables the realization of digital logic gates. The Fano metasurface MEMS-based is shown in Figure 3(c). The MEMS-integrated metasurface has the advantages of low power consumption, large modulation range, and full band regulation from visible light to terahertz, which is one of the competitive schemes for dynamic light modulation calculation. The true (ON) and false (OFF) states of the far-field Fano resonance amplitude are represented by the binary digits “1” and “0,” respectively. The THz far-field transmission spectrum shows the XOR logic properties of the MEMS resonator in different metastable structural states (00), (10), (01), and (11). Voltage inputs (V1 and V2) applied to a single resonator can be programmed using sequential trigger bits {0,1} to control the drive heights (up/down) of SRR-1 and SRR-2, respectively. However, MEMS-based compact photonic devices are usually more complex in the process of structural design and characterization, and the component processing is also very challenging, and the cost is high.

#### 3.1.5 Liquid crystals (LCs) nano-antenna

The combination of liquid crystals (LCs) and metasurfaces can also play its unique advantages in optical computing. LCs are composed of molecules with different orientations that can be controlled by external stimuli, such as electric fields or heating [129]. Therefore, LCs are often used as ambient materials in metasurfaces [130]. With the change of phase, the refractive index and dielectric constant of LCs also change. Compared with crystal materials, semiconductor materials, metal materials, and metamaterials, LCs have great flexibility in material design [131], [132]. The versatility of LCs have also led to its widespread application in combination with metasurface nanophotonic devices. A LCs-based tunable scheme is proposed to achieve tunable edge enhancement images based on computational metasurface as shown in Figure 3(d) [72].When light waves pass through the metasurface, the metasurface can perform spatial difference operations. This optical differential operation can be achieved by the interaction between two orthogonal polarization components. By adjusting the external voltage applied to the liquid crystal phase plate, the different phase delay between the two orthogonal polarization components is introduced. Although the edge-enhanced image of the scheme is only one-dimensional, it can quickly switch between the bright field image and the edge image. Therefore, it has a broad application prospect in high-contrast microscopy. Trevon Badloe et al. designed a dual-mode metalens system integrated with LCs that can electrically switch between bright-field imaging and edge-enhanced imaging modes at the millisecond level. The design is integrated into the same single flat optical device, significantly reducing the size, bulk, and weight of the system [133].

With the increasing demands for refined photoelectrical devices, the liquid crystal material gradually reveals a lot of deficiencies. The response time of the liquid crystal device is usually in the number of ten milliseconds, which is difficult to match with some fast response photoelectrical components. Secondly, because the liquid crystal itself is a large molecular material, its molecular weight can reach 200∼500 g/mol, the electrode spacing and pixel size of the reported liquid crystal metasurface are usually large, and it is difficult to reach the sub-wavelength scale. In addition, the liquid crystal device still has shortcomings in the aspects of self-generating thermal response, beam deflection velocity, and large angular deflection efficiency [134], [135]. Nevertheless, the regulatory capability of liquid crystal still occupies an important position in the application of dynamic active photonic devices, and it is a material that needs to be continuously explored in the future of metasurface.

### 3.2 Main methods of optical computing

In recent years, with the rapid development of artificial intelligence (AI), such as AlphaGo, ChatGPT, Midjourney, etc., intelligent equipment and devices have gradually occupied a larger market. However, there is still a problem of excessive information density explosion and energy consumption, which limits the development and application of AI [136]. Therefore, the development of alterative computing technology path has become an urgent requirement. In this way, optical computing has become an important breakthrough direction. The mainstream optical computing methods include analytical methods, optimization algorithms, neural network algorithms, etc.

#### 3.2.1 Analytical method

The Sobel operator is a popular edge detection method with low algorithm complexity, which can be used to calculate the image gradient approximation [137], [138] and edge detection [139], [140]. In a digital image *f* (*x*, *y*), where (*x*, *y*) represents Cartesian spatial coordinates, where *G*_*x*_ and *G*_*y*_ are the gradient components, respectively, in *x* and *y* directions. *G*_*x*_ and *G*_*y*_ are obtained by convolution of the image using the Sobel operator, where *G*_*x*_ = *f*(*x*, *y*) ⊗ *I*_*x*_, *G*_*y*_ = *f*(*x*, *y*) ⊗ *I*_*y*_. The Sobel operator uses a 3 × 3 convolution kernel in both horizontal and vertical directions, and *I*_*x*_ and *I*_*y*_ are shown in Table 1.

**Table 1:** Sobel operators.

*I*_*x*_			*I*_*y*_		
−1	0	1	−1	−2	−1
−2	0	2	0	0	0
−1	0	1	1	2	1

The convolution operation results are obtained by using the horizontal operator and vertical operator template and the pixels on the image. An appropriate threshold (TH) is compared with G, where, $G=\sqrt{G_{x}^{2}+G_{y}^{2}}$ [141]. When TH ≥ G, the edge of the image is determined. Otherwise, this pixel is ignored as a nonedge pixel. However, the edges of most images are complicated, so the calculation of image edges using only horizontal and vertical Sobel operators will cause a large error.

Compared with the first-order Sobel operator, the Laplacian operation as an isotropic second-order differential is an essential mathematical calculation in various fields of physical science and engineering [142], [143]. The optical analog Laplacian is regarded as an effective tool for solving complex and large-scale mathematical problems [144].

The optical analog computing of Laplace operation for a 2D spatial function can be expressed in the Cartesian coordinate as:

(4) $$\nabla^{2}E\left(x,y\right)=\partial^{2}E\left(x,y\right)/\partial x^{2}+\partial^{2}E\left(x,y\right)/\partial y^{2},$$

where, ∇^2^ is the Laplacian operator, and *E*(*x*, *y*) is the electric field distribution of the input signal. So, the Laplace operation on the optical field is:

(5) $$\nabla^{2}E\left(x,y\right)=-k^{2}E\left(x,y\right),$$

where $k^{2}=k_{x}^{2}+k_{y}^{2}$ and is the in-plane wave vector. So, the optical transfer function (OTF) of the Laplacian should be $T\left(k\right)=-k^{2}$ , which is a quadratic function of the in-plane wave vector of the input light field. Therefore, for normal incidence, the transmittance of the Laplacian should be zero, and as the incidence angle increases, the transmittance should increase.

**Figure 4:**
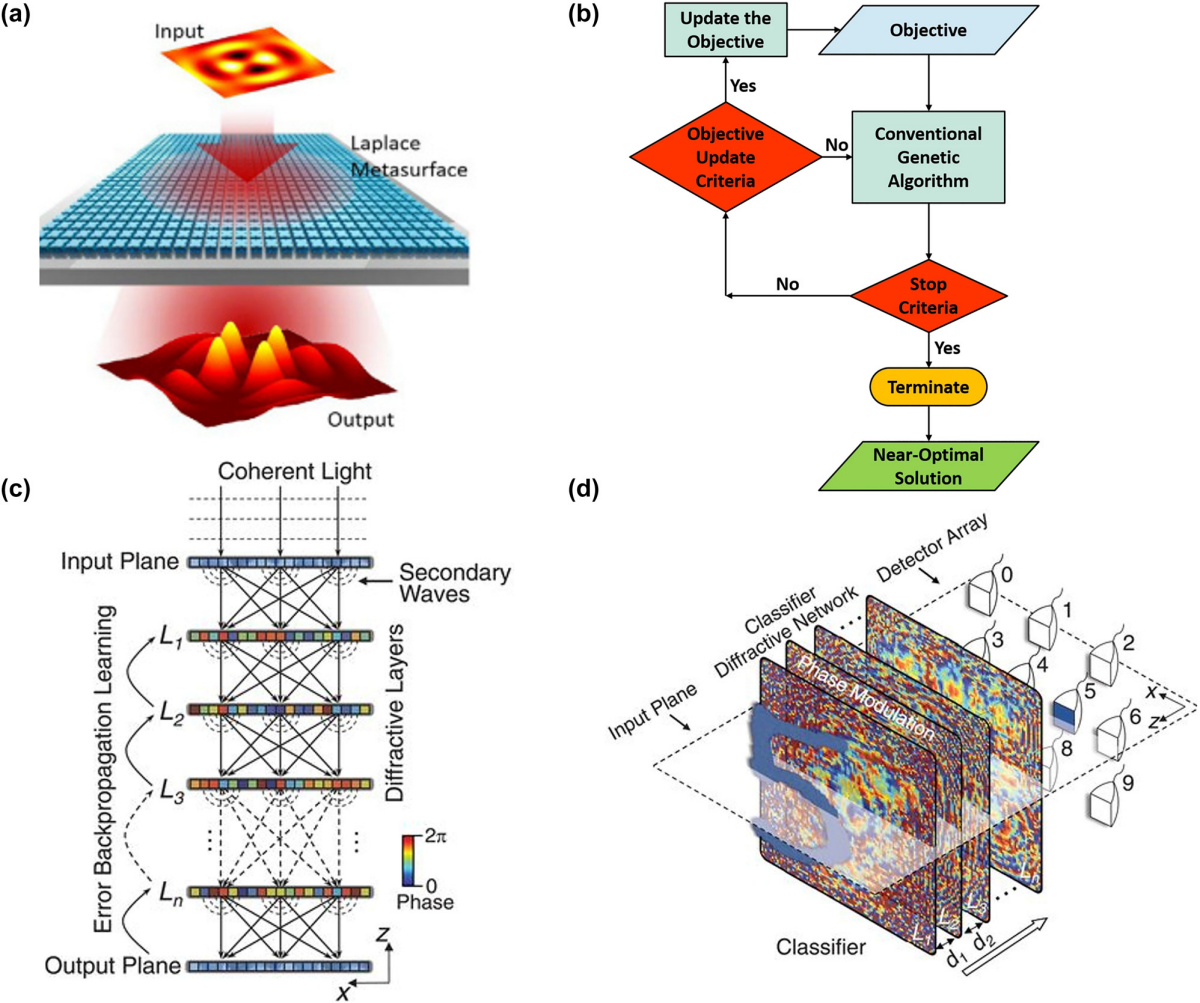
Optical computing methods. (a) Illustration of a dielectric metasurface transforming an input 2D spatial function to another function as a Laplace operator. (b) The schematic of optimize algorithm. (c) Optical system of D^2^NN. *L*_1_, *L*_2_, *L*_3_, and *L*n: first, second, third, and *n*th diffractive layer. (d) A 3D-printed D^2^NN successfully classifies handwritten input digits (0–9) based on 10 different detector regions at the output plane of the network, each corresponding to one digit. (a) Reproduced with permission [69]. Copyright 2021, Optica Publishing Group. (b) Reproduced with permission [148]. Copyright 2018, Nature Publishing Group. (c)–(d) Reproduced with permission [155]. Copyright 2018, American Association for the Advancement of Science.

Many optical computing metasurfaces based on Laplacian operators have been reported. A. Saba et al. proposed a second-order differentiator based on an all-dielectric metasurface [145]. Using the grating guided wave mode, the device can realize the two-dimensional Laplacian operator operation for *S*-polarized incident waves. When the incident light passes through the Laplacian metasurface, the incident light field is automatically processed, as shown in Figure 4(a) [69]. By exciting the quasi-bound state in the continuum (BIC), an optical transfer function for nearly perfect isotropic second-order differentiation has been obtained with a higher spatial resolution. The Laplace calculation result can be obtained quickly by recording the output optical field. Specifically, a two-dimensional Laplace operator operating in visible and near-infrared bands was designed. Accordingly, an image is illuminated through a Laplacian metasurface, and image edges can be obtained at the output plane [146]. The experimental results show that the edge detection resolution of the device was as high as 4 μm. By integrating the photonic crystal-based differentiator with the metasurface lens, a compact differential computing system can be constructed.

#### 3.2.2 Optimization algorithm

Genetic algorithms (GAs) are stochastic search optimizers that are based on the concepts of evolution and natural selection [147], [148], shown in Figure 4(b). GAs are inherently parallel algorithms. Therefore, GAs are widely used in dealing with multidimensional function domain, discrete solution domain problem, and nondifferentiable objective function. Furthermore, a metasurface based on an adaptive genetic algorithm (AGA) is proposed, capable of solve the complex problems in the field of optics. It can diffract TE and TM excited waveguide modes in any chosen direction. These applications demonstrate the advantages of combining metasurface and AGA technology.

#### 3.2.3 Neural network optimization algorithm

Deep learning is a crucial tool for performing advanced inference tasks at present [70], [149], [150], [151], [152], [153], [154]. An all-optical diffractive deep neural network (D^2^NN) architecture is introduced as a physical mechanism for performing machine learning [155]. The architecture can accomplish various functions based on the design of passive diffraction layers using deep learning. The D^2^NN is placed vertically in the direction of light propagation by the multilevel diffraction optical element (DOEs) with preset distribution. The incident light field propagates forward in free space through DOEs. All pixel values (phase-only, amplitude-only, or complex-amplitude) are optimized by an error backpropagation algorithm similar to their counterparts in deep learning. The spatial light intensity distribution in the input plane and output plane corresponds to the input vector and output vector, respectively, as shown in Figure 4(c). The information processing capability of the D^2^NN system depends on the number of diffraction layers [156]. The whole optimization system is linearly transformed from the input light field to the output light field. As an example, D^2^NN recognition of handwritten input “5” is demonstrated in Figure 4(d), where the blue dotted squares represent the trained detector regions for each digit. This architecture can be used as a linear classifier and has been proven to be able to optically classify handwrites digit images in the Modified National Institute of Standards and Technology (MNIST) dataset and fashion product images in the Fashion-MNIST dataset with moderately high accuracy [157].

### 3.3 Metasurface optical computing applications

#### 3.3.1 Edge detection and enhancement

An image is the basis component of human vision. The image edge is defined as the boundary region where the brightness, phase, and polarization of the target image undergo significant changes. Generally, human eyes are more sensitive to the edge information than the flat area. Hence, the image edge information occupies a critical position in human and machine vision.

**Figure 5:**
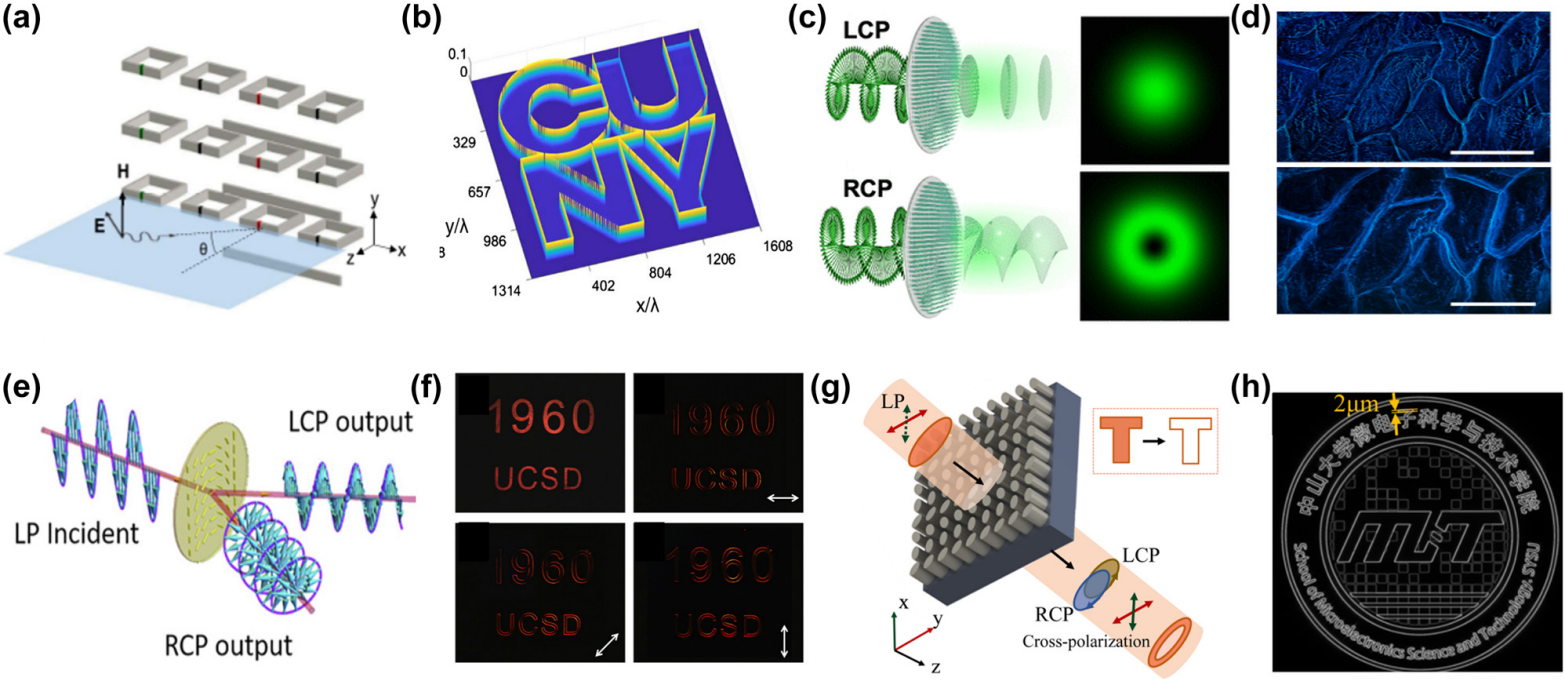
Edge enhancement metasurfaces. (a) Schematic of a metasurface consisting of a periodic array of resonant particles in the *x*–*y* plane, formed by split ring resonators (SRR) parallel to the *x*–*z* plane (magnetic dipole moment parallel to the *y* axis). (b) The output image used to illuminate a metasurface with unpolarized light. (c) Schematic diagram of metasurface edge detection. (d) Images of the undyed onion epidermal cells with a 20× objective lens. Traditional bright-field images captured with LCP incident light at the wavelength of 480 nm. Spiral phase contrast images captured with RCP incident light at the corresponding wavelengths. Scale bar: 100 μm. (e) When PB phase gradient metasurface is illuminated by collimated LP beam, two separated LCP and RCP beam components are observed. (f) Original image without edge detection. Different portion of the edge shows up for different metasurface orientations. Arrows indicate the edge-detection direction. (g) Schematic diagram of metasurface edge detection of a 2D optical differentiator. (h) Output edge image with metasurface period of 27.36 µm. (a)–(b) Reproduced with permission [159]. Copyright 2018, American Physical Society. (c) and (d) Reproduced with permission [161]. Copyright 2020, American Chemical Society. (e) and (f) Reproduced with permission [162]. Copyright 2019, National Academy of Sciences.(g)–(h) Reproduced with permission [71]. Copyright 2023, Optica Publishing Group.

The technique of optical edge detection was developed by Zelnick in 1942 to improve the cell’s contrast [158]. Then, by introducing slow periodic spatial modulation on the metasurface, the nonlocal response can be significantly improved. A spatially modulated metasurface with a suitably designed nonlocal response is proposed for performing different types of mathematical operations on optical signals [159]. The metasurface consists of an array of periodically resonating particles in the *X*–*Y* plane, as shown in Figure 5(a), which consists of split-ring resonators (SRRs) parallel to the *X*–*Z* plane (magnetic dipole moment parallel to the *y*-axis) [160]. The metasurface is excited by TM polarized waves propagating in the *x*–*z* plane. The metasurface with broken *x* and *z* symmetry results in asymmetric responses with respect to ±*k*_*x*_, achieving the required first-derivative operation. However, the disadvantage of this method is that for *x*-polarized illumination, the metasurface allows detection along the *x*-axis but not along the *y*; it performs edge detection only for TM waves. Nevertheless, many scenarios in practical applications use unpolarized light to illuminate the image, as shown in Figure 5(b). The nonlocal metasurfaces perform basic mathematical operations, paving the way toward fast and power-efficient ultrathin devices for edge detection and optical image processing.

By designing the different photon spin response characteristics, the dual-path spin vortex PB-metasurface is proposed in Figure 5(c) [161]. When the LCP incident light is incident on the metasurface, the light is generated with a constant phase profile and Gaussian intensity distribution, and the rotation of the incident polarization is flipped to RCP. For RCP incident light, the rotation of polarization is flipped to LCP. And the light produced has a spiral phase distribution and a donut-shaped intensity distribution. Moreover, the system can easily detect samples with a small refractive index difference from the background environment, such as biological cells, as shown in Figure 5(d). However, the metasurface system is hard to calculate such as first-order differentiation. In addition, edge enhancement methods based on the vortex phase cannot precisely control edge resolution, resulting in limited detection accuracy.

An optical spatial differentiator is proposed, which consists of a designed PB phase metasurface inserted between two orthogonal linear polarizers [162]. As shown in Figure 5(e), when a linear-polarized plane wave is incident on a PB phase gradient metasurface with phase $\varphi \left(x,y\right)=\frac{\pi x}{\Lambda }$ , the LCP and RCP components gain additional phase +2*φ* and −2*φ*. It can be understood that the metasurface divides the input horizontally polarized beam into LCP and RCP, and there is a certain lateral displacement of the two beams traveling in different directions. The edge portion contains only the LCP or RCP components, so after passing through the vertical analyzer, only the outer edge information is left. Furthermore, radial edge detection based on metasurface can be easily extended to 2D edge detection by changing the one-dimensional phase gradient $\varphi \left(x,y\right)=\frac{\pi x}{\Lambda }$ to a radial phase gradient $\varphi \left(x,y\right)=\frac{\pi \sqrt{x^{2}+y^{2}}}{\Lambda }$ . As shown in Figure 5(f), an image of “1960 UCSD” without using the analyzer is represented. Therefore, the system needs to be used with an analyzer in most cases. Similarly, Figure 5(g) shows how the medium metasurface performs two-dimensional edge detection [71]. The highest resolution achieved is 1.7 μm at the period of 27.36 µm (Figure 5(h)), which is close to the diffraction limit of the optical system. Compared to 1000 µm size metasurfaces reported in previous work [163], a higher contrast ratio is obtained with a smaller device size, which is conducive to miniaturization. Meanwhile, elliptical cylinders with simpler structures are adopted, which make it easier to integrate with the CMOS process and facilitate fabrication.

#### 3.3.2 Object recognition

**Figure 6:**
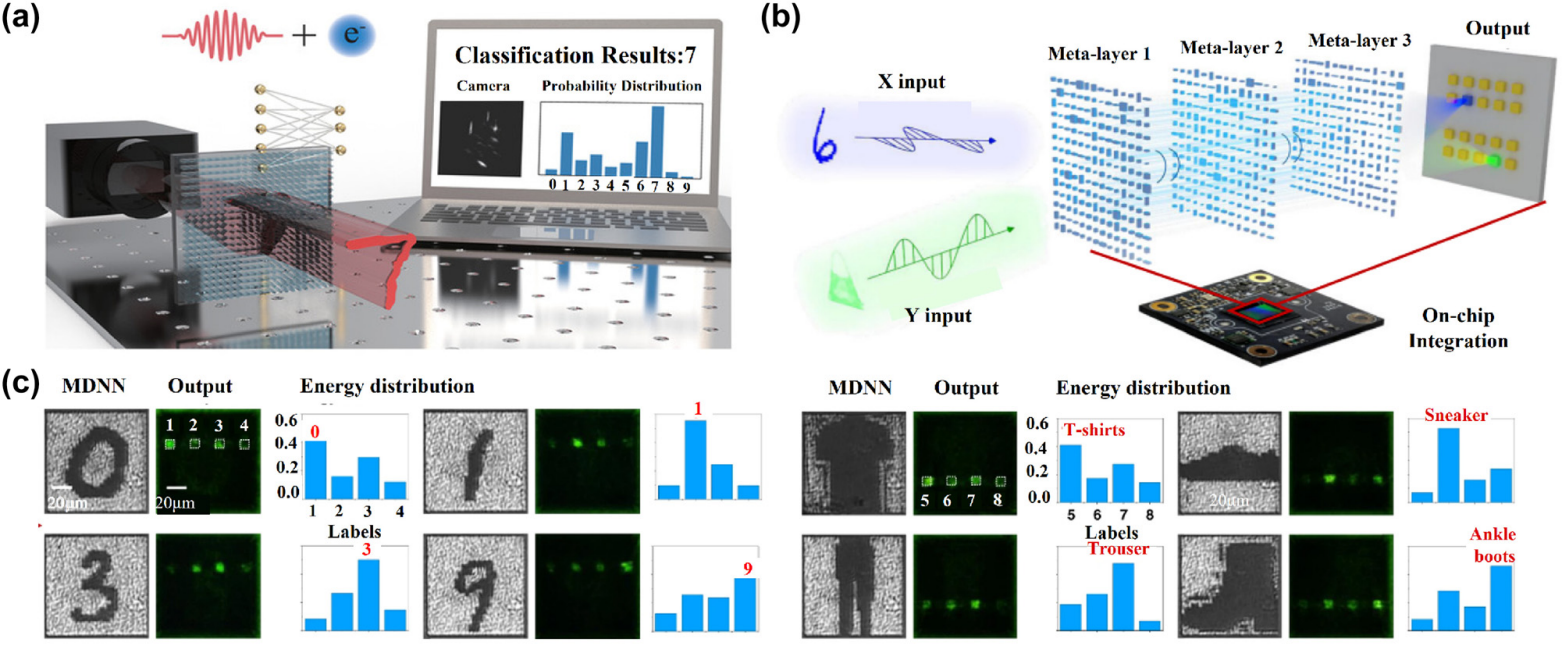
Classification computing metasurfaces. (a) The schematics of the OENN and the corresponding framework. (b) Optical MDNN layout of polarization-dependent object classification. (c) The fabricated MDNN (1st and 4th columns), the output field intensity detected for these four-category classification MDNN in *x*-or *y*-polarization (2nd and 5th columns), and experimentally detected energy distribution (3rd and 6th columns). (a) Reproduced with permission [164]. Copyright 2022, Wiley-VCH. (b) and (c) Reproduced with permission [66]. Copyright 2022, Nature Publishing Group.

Object recognition plays an important role in optical computing fields. A diffraction neural network is proposed to perform machine learning, and this structure can realize various functions of passive diffraction layers designed based on deep learning. The all-optical deep learning framework can analyze and detect features in all-optical images at light speed to realize image classification, such as handwritten digits and fashion products. In 2022, Cui’s group proposes an optical-electronic hybrid neural network (OENN), which consists of a single titanium dioxide (TiO_2_) metasurface and a fully connected electron layer [164]. The schematic diagram of an OENN and the corresponding framework are shown in Figure 6(a). The ultra-compact metasurface extracts the features of the input digits. Then, a low-complexity electronic fully connected layer weights and sums all the features, leading to a final prediction. The combination of nonlocal neural layer and nonlinear transformation greatly expands the capacity of neural networks. Therefore, the classification accuracy of handwritten digit recognition can still be as high as 98.05 % using a single metasurface layer.

All optical diffraction neural networks (ODNN) can perform specific optical computation functions by training the diffraction phase plane to form a cascaded physical network. A polarization-multiplexed metasurface-based diffraction neural network is proposed and integrated with a complementary metal-oxide semiconductors (CMOS) image sensor to create an on-chip multitasking architecture in visible range [66]. As shown in Figure 6(b), the physical model is mainly composed of three parts: the linear polarized plane object wave to be recognized as the input layer; the polarization-multiplexed metasurfaces as the hidden layer; and the image sensor on the CMOS chip as the output layer. The phase distribution of the meta-neurons under each polarization channel is obtained by error backpropagation and stochastic gradient descent algorithm. Furthermore, this configuration can form a multi-channel sensing and computing all-in-one chip architecture. The results of MDNN recognition of different handwritten digits and fashion items in two polarization channels are shown in Figure 6(c). By testing 160 groups of correctly recognized images, it is found that the recognizing accuracy of handwritten digits and fashion products reaches 93.75 % and 95 % in the experiment and simulation, respectively. Although the method can identify numbers and fashion items at the same time, its disadvantage is that it can only identify four numbers rather than all numbers 0–9 at present, as well as the fashion products.

#### 3.3.3 Gesture or movement recognition

**Figure 7:**
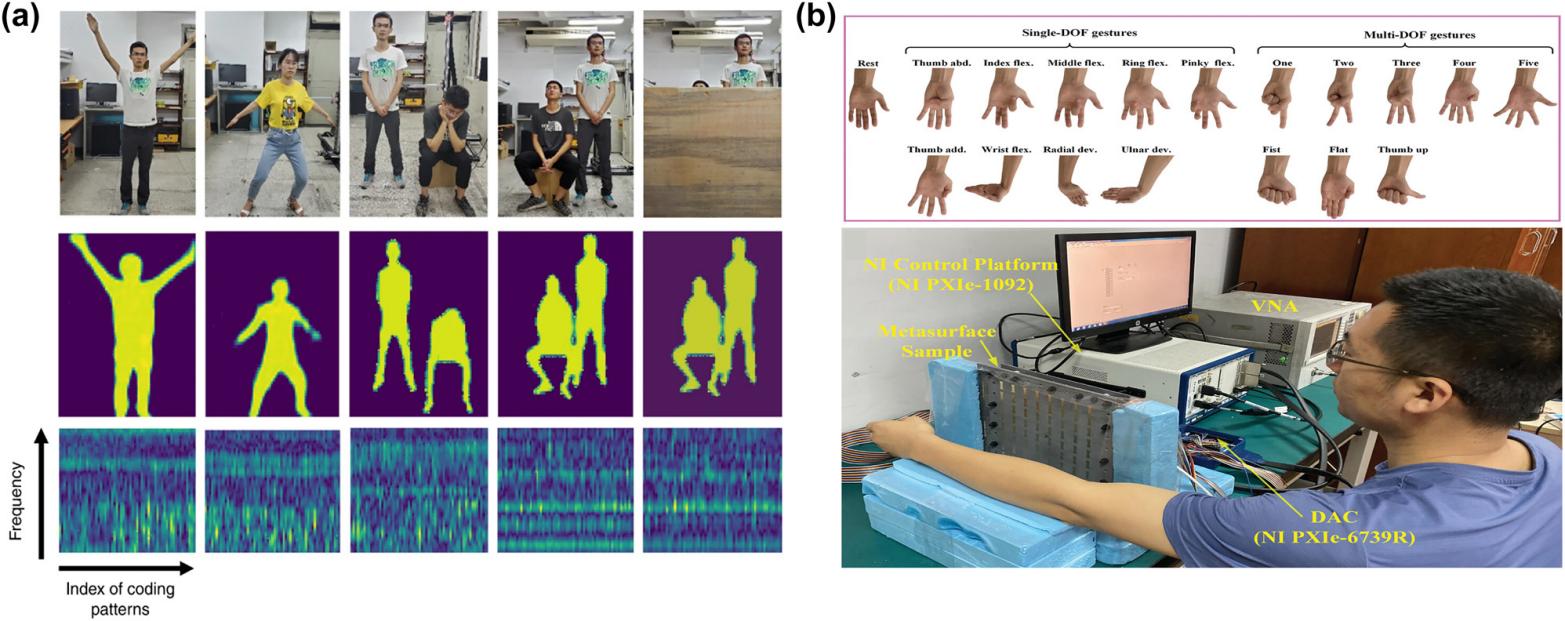
Gesture or movement recognition. (a) Microwave data processing flow by using deep learning CNN cluster. (b) The working principle of the noncontact hand gesture recognition method based on the programmable metasurface sample with a machine learning algorithm. (a) Reproduced with permission [166]. Copyright 2019, Nature Publishing Group. (b) Reproduced with permission [68]. Copyright 2022, Wiley-VCH.

Gesture is a basic human characteristic and an indispensable part of the process of interpersonal communication. The gesture recognition technology has made it possible for man–machine interaction. By mapping commands to outputs for device control, human postures can be explicitly identified as representations of input and processing [101], [165]. Particularly, an intelligent metasurface artificial neural networks system is proposed to convert measured microwave data into an image of the entire human body [166]. Figure 7(a) schematically illustrates three building blocks of the data flow pipeline. The microwave data collected by the intelligent metasurface are instantly processed with an imaging convolutional neural network (CNN) to reconstruct the image of the whole human body. The human breathing is identified by the time-frequency analysis of microwave data. It is worth mentioning that the proposed intelligent metasurface system also works well on stray Wi-Fi signals.

Meanwhile, a noncontact gesture recognition method based on transmission programmable metasurface is proposed [68]. As shown in Figure 7(b), the inner forearm is mapped to a 2D plane, where the *x* and *y* axes are parallel and perpendicular to the direction of the arm, respectively. Using dynamic control of electromagnetic focusing in wavefront engineering has more focused spots to obtain comprehensive echo data. Concretely, it is processed by machine learning to realize high-precision noncontact gesture recognition. In the frequency range of 5–6 GHz, echo coefficients under 5 different focal points controlled by a programmable metasurface were recorded for successfully identifying 18 gesture and wrist movements. Compared with the above system [166], the types of gestures recognized have been improved. However, there are some drawbacks to the system, such as the need for the arm to remain stable during testing. And the arm needs to be wrapped within 10 cm of the metasurface, which can cause discomfort in practical applications. At present, it is difficult to achieve accurate classification and identification between individuals, so in future studies, more data can be collected from different populations. And design a deep neural network model that may help solve cross-individual problems.

#### 3.3.4 Logical operation

The logical operations demonstrate the key role of electronic computing, which can perform general calculations and possess fast processing speed, low crosstalk, and high throughput. However, the optical logic gates currently reported rely heavily on precise control of the input optical signals, including phase difference, polarization, intensity, and the size of the incident beam. Due to the complexity and difficulty of these precise controls, the output optical logic states may have instability and low-intensity contrast [167]. In addition, lighting devices are considered to achieve the miniaturization of optical logic gates. Therefore, getting rid of these complex controls remain challenging in a compact photonic system and achieving full logic functionality.

**Figure 8:**
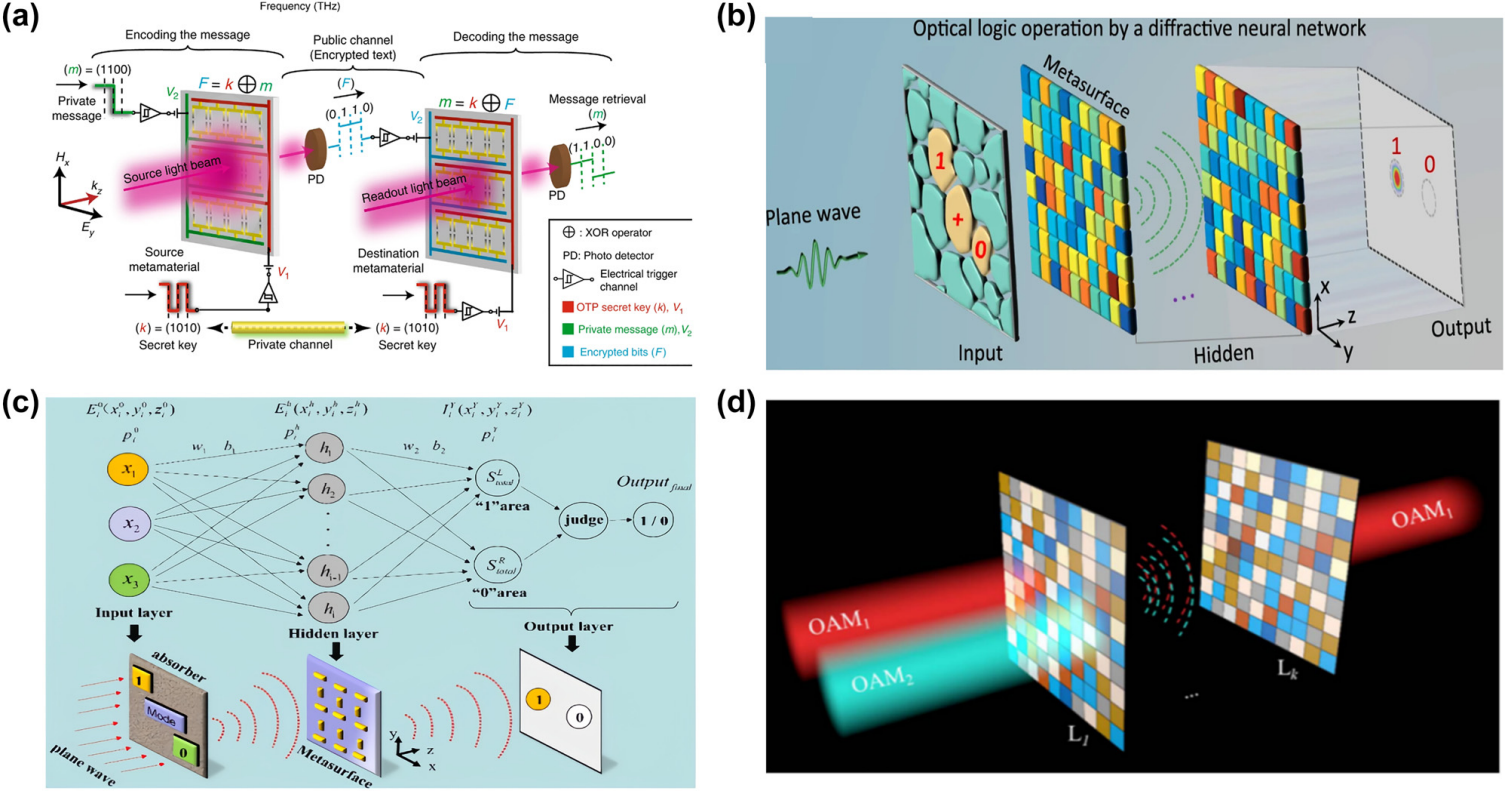
Logical computational metasurfaces. (a) Pictorial representation of realizing the OTP secured wireless communication channel by performing the XOR logic operation to encode the private message (*m*) with the secret key (*k*) and is sent through the public channel as optical signals. The message is retrieved securely (decrypted) at the destination end by performing the inverse XOR operation on the measured optical states (*F*) with the secret key (*k*). (b) Layout of a diffractive neural network for photon-based logic operations. (c) The concept of the FCNN used for logical operation. (d) Schematic of OAM mode logical operation based on ODNN. (a) Reproduced with permission [63]. Copyright 2018, Nature Publishing Group. (b) Reproduced with permission [67]. Copyright 2020, Nature Publishing Group. (c) Reproduced with permission [101]. Copyright 2022, Optica Publishing Group. (d) Reproduced with permission [168]. Copyright 2021, Optica Publishing Group.

A special class of structurally reconfigurable metasurfaces based on micro/nano electromechanical systems (MEMS/NEMS) offers unique advantages in actively manipulating the near field in all three spatial directions by exploiting sensitive changes in micro/nanoscale motion. A reconfigurable MEMS Fano resonant metasurface is proposed with a multi-input–output (MIO) state [63]. It has two independently controlled electrical inputs and an optical read-out at a terahertz frequency to perform logical operations. Similar to the digital XOR logic operation shown in Figure 8(a), when the input voltage states are different, the Fano output is true (*F* = 1), otherwise the Fano output is false (*F* = 0) (when both inputs are true (11) or false (00), the output state *F* = 0). However, MEMS-based metasurfaces are usually more complex in the process of structural design and characterization, and the component processing is also very challenging, and the cost is high.

In addition, the optical logic calculation can also be carried out through diffraction neural networks using plane waves as incident signal [169]. The incident plane wave is spatially encoded by specific logical operations in the input layer. Then, the composite Huygens metasurface decodes by the hidden layer as shown in Figure 8(b) [67]. After well training, the coded light is scattered through a carefully designed metasurface to one small designated region of the output layer. The detected spot provides information on the output logic state, one representing the logic state “1” and the other representing the logic state “0.” Concretely, with a trained diffraction neural network, three kinds of logical operations (NOT, OR, AND) can be implemented at microwave frequencies. The disadvantage of this system is that only three kinds of logical calculation can be performed, and the types of logical calculation need to be improved.

Since the output result of the optical logic operator has only two states of “1” and “0,” this is very similar to the binary classification in deep learning architectures [170]. Therefore, optical logic operators can be constructed according to relevant machine learning theories. Similarly, a compact logical calculator based on a phase metasurface can also be realized by mapping nodes in a trained fully connected neural network (FCNN) [101]. When an incident wave illuminates a selected operating region of the metasurface, the corresponding cell is activated. Then, the wave scatters to two specified regions presenting a logical state in the output layer. After training the FCNN, the basic logical operation functions (AND, OR, NOT) can be implemented on the same metasurface, as shown in Figure 8(c). In addition, the artificial neural network with only one hidden layer is realized by using a single metasurface. That not only reduces the resources and time of training the network but also provides the route for the realization of compact photonic processors and integrated optical computing systems. The neural network utilizes only two optical layers, which provides remarkable improvements in training speed and computational resource usage.

Beyond that, the OAM mode as a kind of freedom is modulated by optical diffraction neural networks (ODNNs) [155], [171], [172] demonstrates good information processing light field modulation and capabilities [168]. The logical operation principal diagram of OAM mode based on ODNN is shown in Figure 8(d). The model solves the linear response of multiple optical fields through the connection of multi-layer phase and amplitude. It accurately realizes seven basic binary logic operations and half adder of AND, OR, NOT, NAND, NOR, XNOR, and XOR gates in simulation. This method not only save the physical properties of the input beam but also can effectively complete and extend to multiple logical operations, which provides a potential solution for the practical application of optical digital computing. The disadvantage is that the logic gate is realized through the cascade metasurface, which requires high requirements in experiment and manufacture.

#### 3.3.5 Mathematic function computation

A mathematic function can be decomposed into linear combinations of basic functions. Shi et al. designed an all-silicon monolayer coded metasurface grating to realize addition and subtraction operations [173]. Similarly, simple mathematical operations through metasurfaces have been implemented in recent years [174], [175]. Furthermore, Zhao et al. propose a compact metasurface-based platform driven by deep learning that is capable of performing four basic trigonometric operations at the speed of light [176]. The advantage is that the four trigonometric operations of EM based can be realized under specific input values, and the optimized visual output makes the operation results clear and recognizable. Furthermore, only one hidden layer is used in the diffraction neural network, which greatly reduces the resources and time required to train the network and improves the integration with other photonic systems. The proposed concept work can provide breakthrough inspiration for other optical computing work such as ultra-fast photonic signal processors.

**Figure 9:**
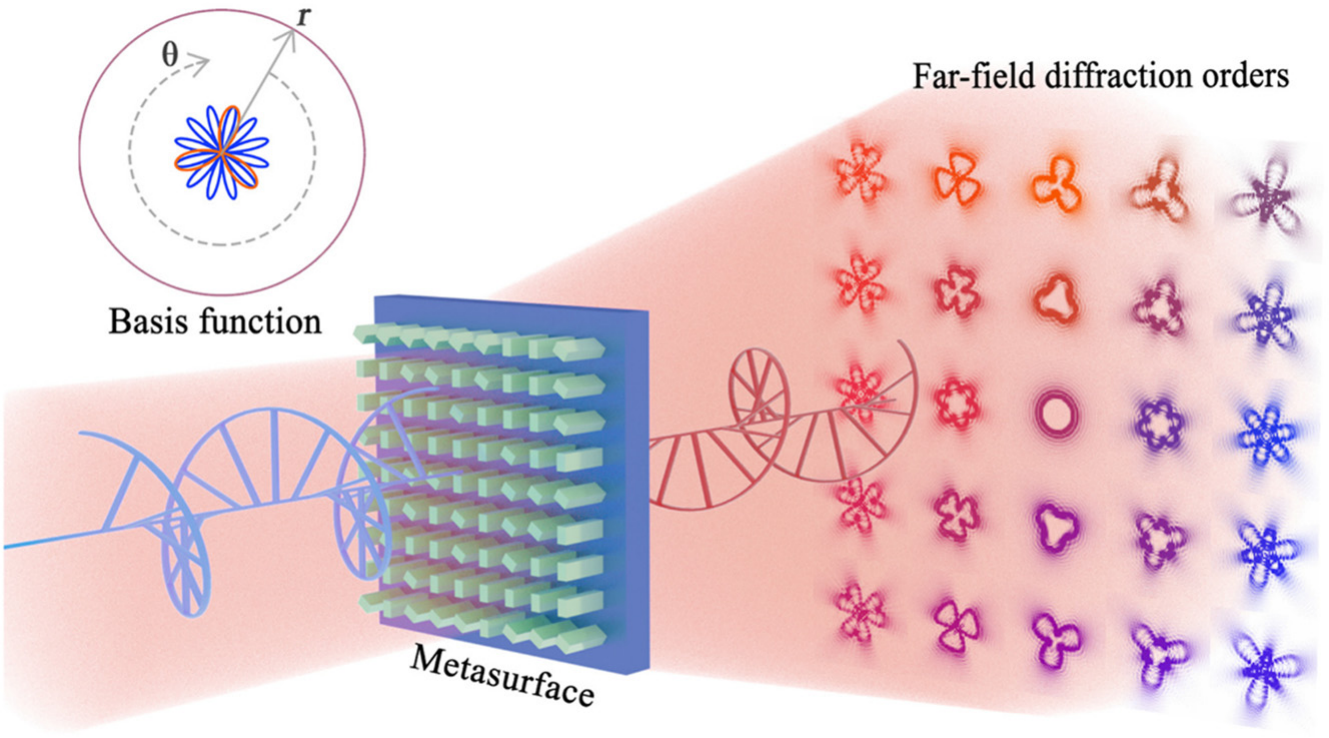
Scheme of DVM based on dielectric geometric metasurface. Diffraction channels (5 × 5) with different intensity patterns of GVBs under orthogonal circular polarization input and output condition. The basis function curves of differential adder and subtractor. Reproduced with permission [177]. Copyright 2022, American Association for the Advancement of Science.

The geometric pattern is from a finite number of basic patterns, thus also enabling beam shaping. Figure 9 graphically shows the generation of diffractive-multiplexed generalized vortex beam (GVB) arrays based on dielectric geometry Dammann vortex metasurface (DVM) [177]. The spatial intensity characteristics of each diffraction stage are no longer the traditional toroidal profile vortex beam, but a free-form distribution carrying OAMs. Through the Taylor series expansion of the vortex grating function, the azimuth phase distribution of each diffraction order can be obtained. Specifically, different configurations of the GVB are generated in different diffraction orders. Thus, the field pattern representing a linear combination of preset basis functions is encoded on the DVM, which can generate GVB arrays with arbitrary angular phase differential design flexibility. Therefore, the generated GVB beams differ from each other in different spatial diffraction orders rather than the regular rings. It provides an optical representation of the features of a mathematical calculation. Such a DVM could be a gateway to explore various optical computing applications.

#### 3.3.6 On-chip optical computing

Optical computing has been demonstrated to enable significant improvements in terms of processing speeds and energy efficiency. Traditional photonic integrated circuit (PIC) devices based on optical waveguides are usually bulky and lack the ability to fully manipulate the light wave at the sub-wavelength scale for optical computing [178]. In recent years, researchers have made a preliminary exploration into the realization of optical computation through on-chip metasurfaces. On-chip metasurfaces build bridge waveguide optics and free-space optics.

**Figure 10:**
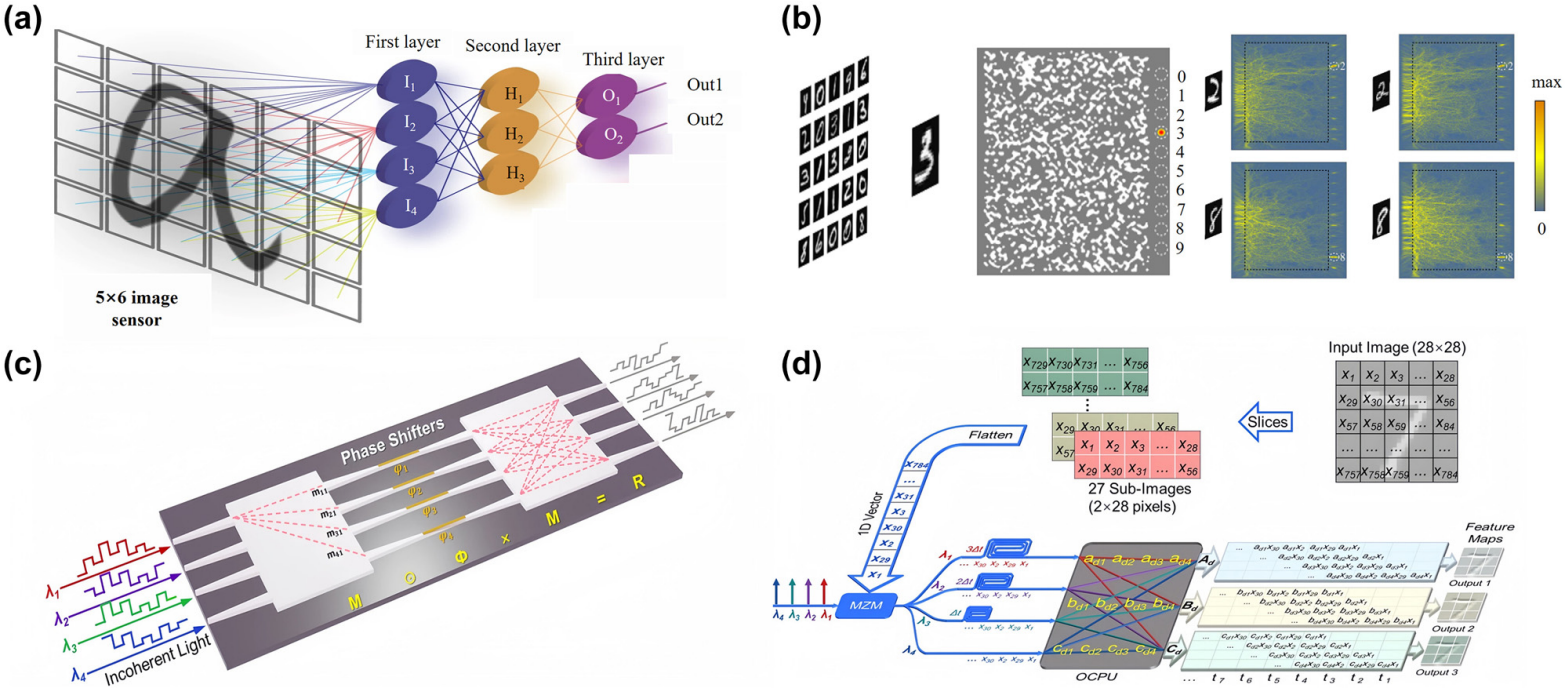
On-chip optical computing metasurfaces. (a) Schematic of the on-chip PDNN structure. (b) NNM trained to recognize handwritten digits. (c) Structure diagram of OCPU. (d) The OCPU simultaneously performs three different groups of convolutional operations using incoherent light. (a) Reproduced with permission [180]. Copyright 2023, Nature Publishing Group. (b) Reproduced with permission [182]. Copyright 2019, Optica Publishing Group. (c)–(d) Reproduced with permission [183]. Copyright 2023, Nature Publishing Group.

A one-dimensional high-contrast transmit array (HCTA) lens defined on a silicon substrate on a standard insulator is proposed, which is a high transmission (<1 dB loss) and above 200 nm bandwidth [179]. By designing 1D HCTA, ultra-short, low-loss, and wide-band mode size converters and meta-systems are obtained to perform Fourier transforms and spatial differentiation. In addition, the cascaded HCTA is used to demonstrate the metasystem functions of Fourier transform and differentiation. The metasurfaces optical computing system has the potential to realize on-chip optical transformation and mathematical operation. Moreover, an integrated end-to-end photonic deep neural network (PDNN) is proposed in Figure 10(a) [180]. The PDNN performs image classification by directly processing the optical waves impacting an array of on-chip pixels as neurons. The PDNN chip was experimentally verified for two-class and four-class handwritten letters classification, achieving accuracies higher than 93.8 % and 89.8 %. Directly, clock-free processing of optical data using PDNN eliminates analog-to-digital conversion and the need for large memory modules. So the research also provides faster and more energy-efficient neural networks for the next generations of deep learning systems. In addition, Fu et al. proposed an on-chip optical diffractive optical neural network (DONN) based on a silicon-on-insulator platform that can perform machine learning tasks with high integration and low power characteristics [181]. The on-chip DONN has the advantages of all-optical passive operation and massive-scale neuron integration. The accuracy of DONN on 1-hidden-layer and 3-hidden-layer on Iris plant dataset classification task is 86.7 % and 90 %, respectively. Phase errors are generated during the fabrication process, the testing accuracy of this method without compensation is 56.7 % and 60.0 %, respectively, and its accuracy needs to be further improved.

On the other hand, a nanophotonic neural medium (NNM) has been proposed to enable artificial neural computation in a continuous and layer-free fashion [182]. Figure 10(b) shows the NNM in action where a two-dimensional (2D) medium is trained to recognize grayscale handwritten digits. On the right side of the NNM, the optical energy concentrates in different locations depending on the image’s classification labels. Inside the NNM, the nanostructure generates strong interference and the light is directed to one of 10 output locations, with the output with the highest share of energy intensity classified as inferred. The computational density of the system is much higher than that of free space and on-chip optical neural networks, and the recognizable numbers achieve 0–9 recognition. However, the average accuracy of the system is more than 79 %, and its recognition accuracy needs to be improved.

In addition, a compact on-chip optical convolutional processing unit (OCPU) was manufactured on a low-loss silicon nitride platform to demonstrate its capability for large-scale integration [183]. The structure diagram of the designed OCPU is shown in Figure 10(c), the input vector is simultaneously modulated on the amplitude of four incoherent light waves with the same initial amplitudes via electro-optical modulation. Figure 10(d) shows that the OCPU simultaneously performs three different groups of convolutional operations using incoherent light. The OCPU includes two 4 × 4 multimode interference cells and four phase shifters and simultaneously performs a convolutional operation with three user-defined 2 × 2 real-valued kernels. Dynamic reconfiguration to extract the desired feature images is realized by tuning the four phase shifters (PSs). That’s experimental accuracy of ten-class of MNIST handwritten digits reached 92.17 %. And the proposed OCPU has the potential of large-scale on-chip integration by simply increasing the number of ports and wavelength division multiplexing at each port. Therefore, it provides the development direction for the optical computing of OCPU with higher processing speed and lower power consumption.

## 4 Conclusion and perspective

In summary, we introduce the fundamental modulation principles of metasurfaces, including four key aspects: phase, amplitude, polarization, and frequency. We discuss the recent research progress in metasurface-based optical computation. Two kinds of optical calculation methods, analytical algorithms and optimization algorithms, are discussed. Complementally, we introduce the idea that different metasurfaces are composed of various materials nano-units. These metasurfaces find widespread applications in optical computing, including image edge detection, digital or image classification, logic computing, and on-chip optical computing. Different from other optical computing technologies, metasurface optical computing shows unique advantages in modulating optical fields, such as multidimensional, integrated, thin and light, programmable, and so on. Therefore, metasurfaces open new avenues for optical information processing and optical computing. It is expected to solve the inherent problems of traditional optical systems. It is expected to make optical computing device more lighted and integrated.

To adapt to the development of intelligent photonics in the new period, there will be greater demand for the improvement of computing power with many digital technologies. Benefiting from the unique advantages of photons, optical computing technology based on metasurfaces is expected to build special computing systems with high speed, high computing power, and high energy efficiency. As mentioned earlier, different building units and computational methods can be suitable for very specific tasks with their unique characteristics. For example, the optical system metasurfaces-based using spatial differentiation operation can realize the image edge detection or enhancement more accurately and effectively. As far as edge detection applications are concerned, real objects are often 3D and have certain depth information. How to implement edge detection of complex 3D objects is still a difficult challenge. As well as the realization of digital or item classification, only a category of classification or a small number of classifications cannot be ignored. With the vigorous development of technologies, semantic recognition is also an indispensable technical demand in the future, such as auto-driving, and the recognition of dynamic gestures or scenes. With the fast information transfer speed and deployed 5G/6G networks, spatially coded communication applications contribute to the development of big data, cloud computing, and the Internet of Things (IoT). In addition, applications such as logical computing and beam shaping will broaden the application field of artificial intelligence.

The realization methods of optical computing have evolved from simple analytic algorithms to neural networks. Optical computing has been widely used in different AI models. However, its commercialization and large-scale applications are still not widespread due to various challenges. Although there are still many problems to be solved in the future, current optical computing, especially analog optical computing, has shown the unique potential of light in terms of speed, data parallelization, and power consumption. At present, the optical computing technology based on metasurfaces is developing steadily, but there is still a certain distance from achieving large-scale commercial application. First, light propagation is a linear operation, and deep learning-based light computation also presents challenges in performing complex functions. In addition, since most static metasurfaces cannot change their nano-antenna after manufacturing, backpropagation of deep learning cannot be performed optically completely. The weight of each neuron needs to be calculated, and then the nano-antenna of the metasurface is used to achieve its effect. Further, as light passes through each metasurface, the intensity of the light decays with the multilayer structure. This makes it difficult to build multi-layered optical deep learning networks, which limits more complex network structures. In particular, the key of optical computing to meet the needs of artificial intelligence computing power is to develop parallel modulation/demodulation technology of optical field. The optical computing system based on single or double layer metasurface can only realize some special calculation process and lack the ability of flexible adjustment. Meanwhile, the calculation accuracy of the whole system is still limited by the processing technology of metasurface nano-units. Multilayer metasurface plays an important role in improving the performance of optical neural networks. However, the cascade of multilayer metasurface is also a difficult problem. At present, only two levels of metasurface can be achieved, and it is almost difficult to achieve three-level metasurface. The main problem is that it is difficult to improve the alignment accuracy of the cascade metasurface. In addition, the algorithms and architectures of optical computing based on metasurfaces still need to be expanded and improved to match the progress of existing AI development. Therefore, we believe that more effort needs to be put into addressing the key shortcomings of light and demonstrating the advantages of optical computing over electronic computing in different practical applications.

Metasurfaces are not only being applied to the current devices throughout the electromagnetic spectrum from microwave to optics wavelength but also inspiring many new thrilling applications. Due to the diversity of different material types, the structural units of the metasurface are widely realized by various materials. Although metasurfaces of various structural units are emerging in the field of optical computing, there is no single perfect structural unit that can meet the needs of these more advanced industrial applications in every metric. In addition, it can further promote the upgrading of micro/nanofabrication technology, new active materials research, or combine a variety of regulatory schemes to complement each other’s advantages. This may provide new opportunities for better implementation of metasurface optical computing. In the future, it is believed that with the further development of metasurface design methods, the accuracy and speed of metasurface calculation will be further improved. In some fields, it can replace traditional electronic computing equipment and be more widely used.
